# Enhanced Th17-Cell Responses Render CCR2-Deficient Mice More Susceptible for Autoimmune Arthritis

**DOI:** 10.1371/journal.pone.0025833

**Published:** 2011-10-04

**Authors:** Rishi R. Rampersad, Teresa K. Tarrant, Christopher T. Vallanat, Tatiana Quintero-Matthews, Michael F. Weeks, Denise A. Esserman, Jennifer Clark, Franco Di Padova, Dhavalkumar D. Patel, Alan M. Fong, Peng Liu

**Affiliations:** 1 Department of Medicine, Thurston Arthritis Research Center, University of North Carolina at Chapel Hill, Chapel Hill, North Carolina, United States of America; 2 Division of General Medicine and Clinical Epidemiology, Department of Medicine, University of North Carolina at Chapel Hill, Chapel Hill, North Carolina, United States of America; 3 Department of Biostatistics, University of North Carolina at Chapel Hill, Chapel Hill, North Carolina, United States of America; 4 Novartis Institutes for BioMedical Research, Basel, Switzerland; Università degli Studi di Milano, Italy

## Abstract

CCR2 is considered a proinflammatory mediator in many inflammatory diseases such as rheumatoid arthritis. However, mice lacking CCR2 develop exacerbated collagen-induced arthritis. To explore the underlying mechanism, we investigated whether autoimmune-associated Th17 cells were involved in the pathogenesis of the severe phenotype of autoimmune arthritis. We found that Th17 cells were expanded approximately 3-fold in the draining lymph nodes of immunized CCR2^−/−^ mice compared to WT controls (p = 0.017), whereas the number of Th1 cells and regulatory T cells are similar between these two groups of mice. Consistently, levels of the Th17 cell cytokine IL-17A and Th17 cell-associated cytokines, IL-6 and IL-1β were approximately 2–6-fold elevated in the serum and 22–28-fold increased in the arthritic joints in CCR2^−/−^ mice compared to WT mice (p = 0.04, 0.0004, and 0.01 for IL-17, IL-6, and IL-1β, respectively, in the serum and p = 0.009, 0.02, and 0.02 in the joints). Furthermore, type II collagen-specific antibodies were significantly increased, which was accompanied by B cell and neutrophil expansion in CCR2^−/−^ mice. Finally, treatment with an anti-IL-17A antibody modestly reduced the disease severity in CCR2^−/−^ mice. Therefore, we conclude that while we detect markedly enhanced Th17-cell responses in collagen-induced arthritis in CCR2-deficient mice and IL-17A blockade does have an ameliorating effect, factors additional to Th17 cells and IL-17A also contribute to the severe autoimmune arthritis seen in CCR2 deficiency. CCR2 may have a protective role in the pathogenesis of autoimmune arthritis. Our data that monocytes were missing from the spleen while remained abundant in the bone marrow and joints of immunized CCR2^−/−^ mice suggest that there is a potential link between CCR2-expressing monocytes and Th17 cells during autoimmunity.

## Introduction

Th17 cells are IL-17-producing T helper effector cells that are distinct from Th1 and Th2 cells, and from regulatory T (Treg) cells. Th17 cells have been suggested to mediate inflammation and play a key role in the pathogenesis of tissue-specific autoimmune diseases including experimental autoimmune encephalomyelitis (EAE), collagen-induced arthritis (CIA), and psoriasis [1], [2]. Specifically, studies found that mice lacking a Th17 cell-promoting cytokine IL-23 were resistant to EAE or CIA [3], whereas mice deficient for the classic Th1 cell cytokine IL-12 or IFN-γwere more susceptible to these diseases [4], [5]. Adoptive transfer of Th17 cells was shown to be required for EAE [6]. Furthermore, gene targeted deletion of IL-17 or treatment with a neutralizing anti-IL-17 antibody results in CIA resistance [7], [8]. These results suggest that Th17 cells are potent inducers of autoimmune disorders. Since the IL-17 receptor is expressed on epithelial and parenchymal cells, Th17 cells are thought to promote tissue inflammation by producing IL-17 to stimulate the production of IL-6, IL-1, tumor necrosis factor (TNF), and other proinflammatory factors [9]. In humans, anti-IL-17A antibodies have shown positive clinical responses and good safety in patients with active rheumatoid arthritis (RA) in randomized, double-blinded proof-of-concept trials [10], [11], suggesting that targeting of IL-17A is a promising therapeutic approach against human autoimmune disease, such as RA.

CCR2 is a chemokine receptor for monocyte chemoattractant protein-1 (MCP-1 or CCL2) and important for monocyte trafficking toward sites of inflammation, a process that is critical for autoimmune diseases like rheumatoid arthritis (RA) and CIA [12]. Since CCR2 is highly expressed on joint infiltrated monocytes/macrophages in RA patients and CIA animals [13], targeted inhibition of CCR2 was initially thought to be a promising therapeutic strategy for the treatment of RA. However, clinical trials using CCR2 or CCL2 neutralizing antibodies did not show efficacy [14], [15]. Application of anti-CCR2 during disease progression or deletion of Ccr2 genes (CCR2^−/−^) unexpectedly induce exacerbated CIA in mice [13], [16]. Although our previous study and others show that inhibiting CCR2 is beneficial to other inflammatory diseases, such as vascular inflammations [17], [18], the mechanism underlying the CCR2 paradox in autoimmune inflammation is not completely understood.

Autoimmune inflammation uniquely involves antigen-specific activation of T cells that leads to subsequent B cell activation and autoantibody formation. Given the specific proinflammatory role of Th17 cells in autoimmune diseases, we hypothesized that skewing of Th17 cells and Th17-cell responses may account for the exacerbated arthritis in CCR2^−/−^ mice. To test this hypothesis, we examined Th17 cells, Th1 cells, Treg cells, and Th17 cell-associated events, such as cytokine profile, autoantibody production, and neutrophil activities, in CCR2^−/−^ mice induced with collagen-induced arthritis. We also treated these animals with an IL-17A neutralizing antibody. Our results demonstrate that both Th17 cells and Th17-cell associated responses are markedly enhanced in immunized CCR2^−/−^ mice and neutralizing IL-17A has an ameliorating effect on the severe autoimmune arthritis seen in CCR2 deficiency. Our data that monocytes decrease in the spleen, but not in the bone marrow and arthritic joints in CCR2^−/−^ mice suggest a potential biological link between CCR2-expressing monocytes and Th17 cells.

## Methods

### CIA induction and evaluations

CCR2^−/−^ mice on DBA/1J background were generated by backcrossing CCR2^−/−^ C57BL/6J mice with wildtype DBA/1J mice (Jackson Laboratory, Bar Harbor, Maine) for 12 generations. 8–10 week-old male CCR2^−/−^ DBA/1J mice (CCR2^−/−^ mice) were used in the experiments and the age- and gender-matched littermates of CCR2^+/+^ DBA/1J mice (WT mice) were used as controls. All mice were bred and maintained in a barrier facility and fed with Prolab RMH-3000 (PMI Nutrition International, Richmond, IN), a normal rodent chow diet. All experimental protocols were in compliance with IACUC (Institutional Animal Care and Use Committee) guidelines and were approved by the IACUC at the University of North Carolina at Chapel Hill (ID: 09-344).

CIA is typically performed with either one or two injections of heterologous type II collagen on a susceptible congenic mouse strain. The main purpose of a second booster is to increase the overall disease severity so that the difference between control mice and gene-deficient or treatment animals that have less disease can be apparent. In our case, CCR2-deficient mice have exacerbated disease that starts from day 14 after one injection, which was prior to the second booster at day 21. Therefore, we chose to utilize single injection to examine effect of CCR2 deficiency on the pathogenesis of CIA. In this study, CIA was induced by immunization with type II bovine collagen (CII, Chondrex, Redmond, WA) emulsified with complete Freund's adjuvant (CFA, Sigma). The immunization and disease evaluation methods were performed according to protocols validated by our group and others with minor revisions [19], [20]. Briefly, CII was dissolved overnight in 0.1 M acetic acid and emulsified in an equal volume of CFA. Mice were immunized with a subcutaneous injection of 100 µl of emulsion containing 100 µg of CII at approximately 1 cm from the base of the tail at day 0. The clinical disease of arthritis was evaluated as follows: 0 = normal; 1 = swelling in 1 joint; 2 = swelling in >1 joint; 3 = swelling of the entire paw. Paw swelling was measured in mm range with a Dial Thickness gauge (Geneva Gage, Albany, OR). Both arthritis scoring and measurement were performed in a blinded manner.

### Flow cytometry

Single cell suspension of the spleen or draining lymph nodes was prepared using a manual tissue homogenizer. Red blood cells (RBC) were lysed with a lysis buffer (0.14 M NH_4_CI, 0.017 M Tris-HCl adjust to pH 7.2). Cells were washed with RPMI containing 2 mM Hepes and 1% bovine serum albumin and passed through a 70 µm nylon cell strainer. Isolation of synovial cells was performed as described previously [21] with minor modifications. Briefly, the hind paws were dissected out and incubated with Collagenase D (Sigma) at 2 mg/ml in PBS at 37°C for 30 minutes. The digestion was stopped by adding 10 mM EDTA and incubated for an additional 5 minutes. Synovial cells were isolated and passed through a 70 µm cell strainer. A CyAn flow cytometer and Summit software (CyAn, Dako) were used to analyze cell surface markers that were stained with fluorophore-conjugated primary and isotype control antibodies (eBioscience and Biolegend). Antibodies used in the experiments included anti-CD3, CD4, CD8, CD45R/B220, CD11b, CD11c, F4/80, Ly6C, Ly6G and their isotype controls that were conjugated with FITC, PE, PerCP, Pacific Blue, Pacific Orange, allophycocyanin (APC), and Alexa Fluor-APC. For intracellular cytokine staining, single cell suspension of draining lymph nodes were cultured in petri dishes pre-coated with anti-CD3 in the presence of anti-CD28 for 6 hours, and Golgi Stop (BD Bioscience) was added for the last two hours. Cells then were harvested and stained with anti-CD4 on the surface, fixed and permeabilized with BD Cytofix/Cytoperm (BD Bioscience), and stained with anti-IL-17A and anti-IFN-γintracellularly. For staining of Treg cells, anti-CD4/CD25 surface stained cells were fixed and permeabilized overnight at 4°C. The following day the cells were blocked for 15 minutes with anti-CD16/CD32, and then stained with anti-FoxP3-PE (eBioscience, San Diego, CA) at 4°C for 30 minutes.

### Analysis of systemic cytokine profile

Systemic cytokine profiles of IL-1β, IL-6, IL-17A, and IFN-γ were determined by Luminex using blood sera collected through submandibular bleeding of CCR2^−/−^ and WT mice with CIA. Sera cytokine levels were measured with a Premixed Beadlyte Kit (Millipore, Billerica, MA) by the Luminex 100 dual laser system and XY Platform (Luminex Corp., Austin, TX), which was controlled by the MasterPlex CT 1.2 software (MiraiBio, Alameda, CA). MasterPlex QT 4.0 system (MiraiBio, Alameda, CA) was used for data analysis and the five-parameter regression formula was used to calculate cytokine concentrations from the standard curves. All luminex assays were performed by the UNC Clinical Proteomics Laboratory. BAFF was detected using a Quantikine Mouse BAFF ELISA Kit from R&D System (Minneapolis, MN) according to manufacture instruction.

### Production of antigen-specific antibodies

The production of antigen-specific antibodies was measured by ELISA using the Mouse Anti-Bovine Type II Collagen IgG Assay Kit (Chondrex, Redmond, WA). Briefly, collagen pre-coated wells were washed and blocked with the blocking buffer and incubated with mouse sera samples with a 1∶10,000 dilution, incubated with an HRP-labeled polyclonal secondary antibody, and developed with the OPD solution. OD values were read at 490 nm on an Emax Precision Microplate Reader and analyzed with SOFTmax PRO, 3.0 (Molecular Devices).

### Quantitative real-time PCR

The expression of IL-17A in the joint tissues was measured by quantitative real-time PCR. Briefly, total RNA was isolated from CIA mouse paws by Trizol followed by a RNA cleaning step using the Qiagen RNeasy Mini Kit (Qiagen, Valencia, CA), and cDNA was generated using the First-Strand cDNA Synthesis Superscript II RT (Invitrogen, Carlsbad, CA). Primers used for the amplification of murine IL-17A, IFN-γ, IL-6, IL-1β, CXCL1, CXCL2, RANKL and 18s rRNA were as follows: IL-17A, 5′-TCTCTGATGCTGTTGCTGCT-3′(forward) and 5′-CTCCAGAAGGCCCTCAGACTAC-3′(reverse); IFN-γ, 5′-ACTGGCAAAAGGATGGTGAC-3′ (forward) and 5′-ACCTGTGGGTTGTTGACCTC-3′ (reverse); IL-6, 5′-TTCCATCCAGTTGCCTTCTT-3′ (forward) and 5′-CAGAATTGCCATTGCACAAC-3′ (reverse); IL-1β, 5′-GGTCAAAGGTTTGGAAGCAG-3′ (forward) and 5′-TGTGAAATGCCACCTTTTGA-3′ (reverse); CXCL1, 5′-CAATGAGCT GCGCTGTCAGTG-3′ (forward) and 5′-CTTGGGGAC ACCTTT TAGCAT C-3′ (reverse); CXCL2, 5′-CCAAGGGTTGACTTCAAGAAC-3′ (forward) and 5′-AGCGAGGCACATCAGGTACG-3′ (reverse); RANKL, 5′-TGTACTTTCGAGCGCAGATG-3′ (forward) and 5′-AGGCTTGTTTCATCCTCCTG-3′ (reverse) and 18s rRNA, 5′-GACCATAAACGATGCCGACT-3′ (forward) and 5′-GTGAGGTTTCCCGTGTTGAG-3′ (reverse). Quantitative real-time PCR was performed in a SYBR Green Master Mix with primers listed above in an iCycler instrument (BioRad Laboratories Hercules, CA). The 2^−ΔΔCt^ method [22] was used for data analysis.

### Anti-IL-17A antibody treatment

CCR2^−/−^ mice were immunized for CIA and divided into two groups of treatment. At day 14 post-immunization, each mouse was given 200 µg of either a murine anti-IL-17A antibody (Novartis, Basel, Switzerland) or an isotype control IgG1 (Biolegend, San Diego, CA) through intraperitoneal (i.p.) injection. Then additional injections were given twice a week for two weeks.

### Statistical analysis

Numerical data presented in the text and figures were expressed as mean ± SEM. Student's unpaired two-tail *t* test was utilized to compare average numbers of cells or percentages between experimental groups. For clinical disease assessment and antibody treatment experiments, arthritis severities were analyzed using a Mixed Effect Model to determine significances over time in arthritis scores and paw swelling. The overall group effect was assessed using a likelihood ratio test (LRT). Analysis was conducted using SAS, v. 9.2 statistical software package (Cary, NC). In all cases, p<0.05 was considered significant.

## Results

### Th17 cells are expanded in the draining lymph nodes and joints of collagen immunized CCR2^−/−^ mice

**Figure 1:**
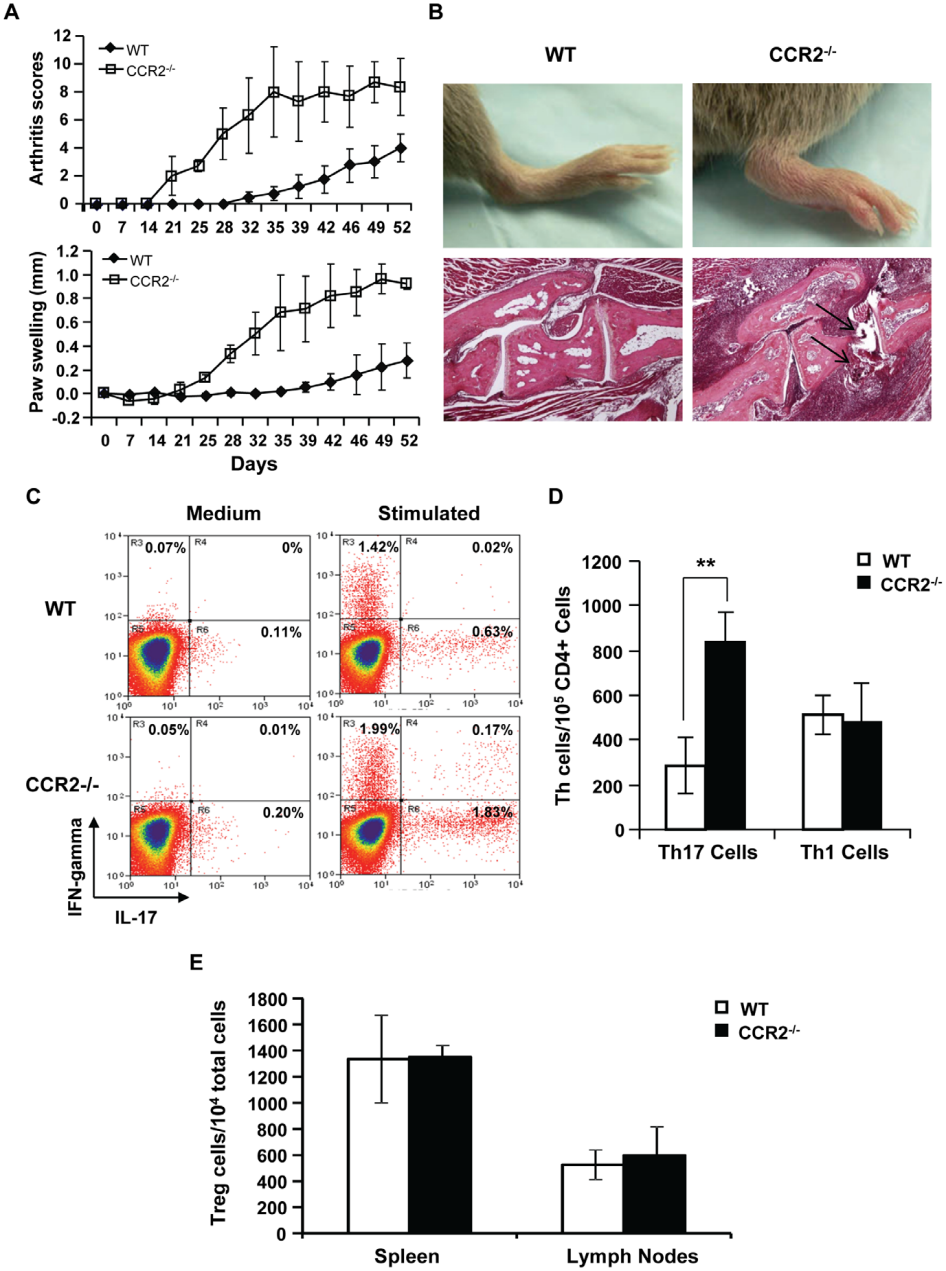
Th17 cells expand in the draining lymph nodes of immunized CCR2^−/−^ mice. **A.** CCR2^−/−^ and WT mice were immunized with bovine type II collagen in CFA at day 0. Arthritis was measured and recorded in arthritis scores and paw swelling over time. These results are representative of one of three experiments with a total of 25 mice. **B.** Representative photos of hind paws (day 26) and their histological sections with the H&E stain (day 48) from WT and CCR2^−/−^ mice are shown. Arrows indicate the extensive inflammation and bone erosion in the joint section of CCR2^−/−^ mice with CIA. **C–D.** Single cell suspension of the draining lymph nodes of immunized mouse was cultured in medium with or without anti-CD3 and anti-CD28 antibody stimulation for 6 hours. They were then harvested and stained with anti-CD4 antibody on the surface and anti-IL-17A and anti-IFN-γantibodies intracellularly. C shows the dot plots of cells gated on CD4^+^ cells in flow cytometry, and D demonstrates the average numbers of Th17 cells versus Th1 cells in the draining lymph nodes of CCR2^−/−^ and WT mice 14 days after immunization, each group contains 5 animals, **p = 0.017. **E.** Treg cells were identified from the spleen and lymph nodes of immunized mice as CD4^+^CD25^+^FoxP3^+^ cells. These data are results of at least three separate experiments.

Both CCR2 and its ligand CCL2 are up-regulated in the synovium of patients with RA, and thus are considered to be proinflammatory in autoimmune inflammation [12]. However, more severe arthritis was developed in CCR2^−/−^ mice that were induced by collagen immunization [13]. Indeed, significant differences were identified over time for CCR2^−/−^ mice in both the clinical arthritis scores (LRT = 26.4, df = 4, p<0.0001) and the paw swelling (LRT-37.5, df = 4, p<0.0001) compared to WT controls (Figure 1A). Disease in CCR2^−/−^ mice occurred 14 days earlier than that in WT controls after one immunization. Between days 42 and 49 after immunization, 100% of CCR2^−/−^ mice and approximately 40% of WT mice received maximal disease, which proceeded to the recovery and resolution phase after this period of time. The severity of arthritis was approximately three times worse during the follow-up time period in CCR2^−/−^ mice with deteriorated histological changes of leukocyte infiltration, bone erosion, and joint destruction (Figure 1 B). These results confirm the previous finding that the absence of CCR2 does not ameliorate but rather aggravates autoimmune arthritis.

Given that autoimmune arthritis is a T cell-mediated disease; and Th17 cells are potent inducers of tissue inflammation in this disease [23], we hypothesized that CCR2 deficiency promotes autoimmune arthritis by facilitating Th17 cell activities. To test this hypothesis we examined Th17 cells in immunized CCR2^−/−^ and wild type mice. We also tested the abundance of Th1 cells and Treg cells. Our data showed that the number of Th17 cells was increased approximately 3-fold in the draining lymph nodes of CCR2^−/−^ mice prior to disease onset (p = 0.017), whereas Th1 cells remained similar between CCR2^−/−^ and WT mice (Figure 1C and D). There was no significant difference of Treg cells in peripheral lymphoid tissues between immunized CCR2^−/−^ and wild type mice (Figure 1E).

**Figure 2:**
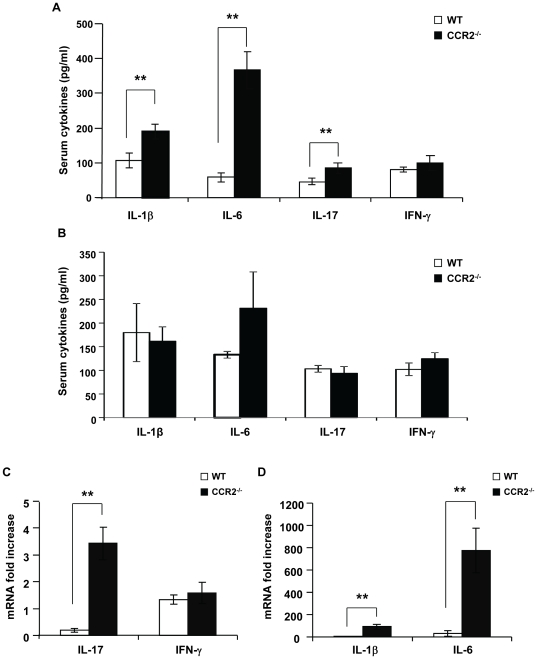
Serum and joint cytokine profiles favor Th17 cell expansion in CCR2^−/−^ mice. **A.** Blood sera were collected at day 26 after immunization from WT and CCR2^−/−^ mice. Levels of cytokines were measured by Luminex assays. Significant differences indicated as asterisks (**) were found in IL-1β (p = 0.01), IL-6 (p = 0.0004), and IL-17 (p = 0.04). Eight animals in each group were used. B. Sera levels of cytokines were measured at day 48 after immunization by Luminex. Four animals in each group were used. **C.** Joint tissues were isolated from the hind paws of mice at day 48 after immunization. The expression of IL-17 and IFN-γ mRNA was measured by quantitative real time PCR in WT (n = 5) versus CCR2^−/−^ (n = 4) mice, **p = 0.009. **D.** Joint tissues were isolated from the hind paws of mice at day 48 after immunization. The expression of IL-1β and IL-6 mRNA was measured by real time PCR in WT (n = 5) versus CCR2^−/−^ (n = 4) mice, **p≤0.02.

Consistently, levels of IL-17A, but not IFN-γ in the serum of CCR2^−/−^ mice were significantly increased (Figure 2A) in the early phase of arthritis. In the meantime, serum IL-6 and IL-1β, which are closely related to Th17 cell differentiation and downstream Th17 cell-mediated proinflammatory responses, were also increased in CCR2^−/−^ mice (Figure 2A). However, such significant elevation of sera cytokines in CCR2^−/−^ mice became less obvious when robust joint disease developed (Figure 2B). Then we went on to test these cytokines in the arthritic joints and found that the expression levels of IL-17A, IL-6, and IL-1β were up-regulated in CCR2^−/−^ mice compared to WT mice (Figure 2C and D). These results suggest that CCR2 deficiency induces increased Th17 cell activities in the generation and development of autoimmune arthritis.

### Increased autoantibody production in CCR2^−/−^ mice

**Figure 3:**
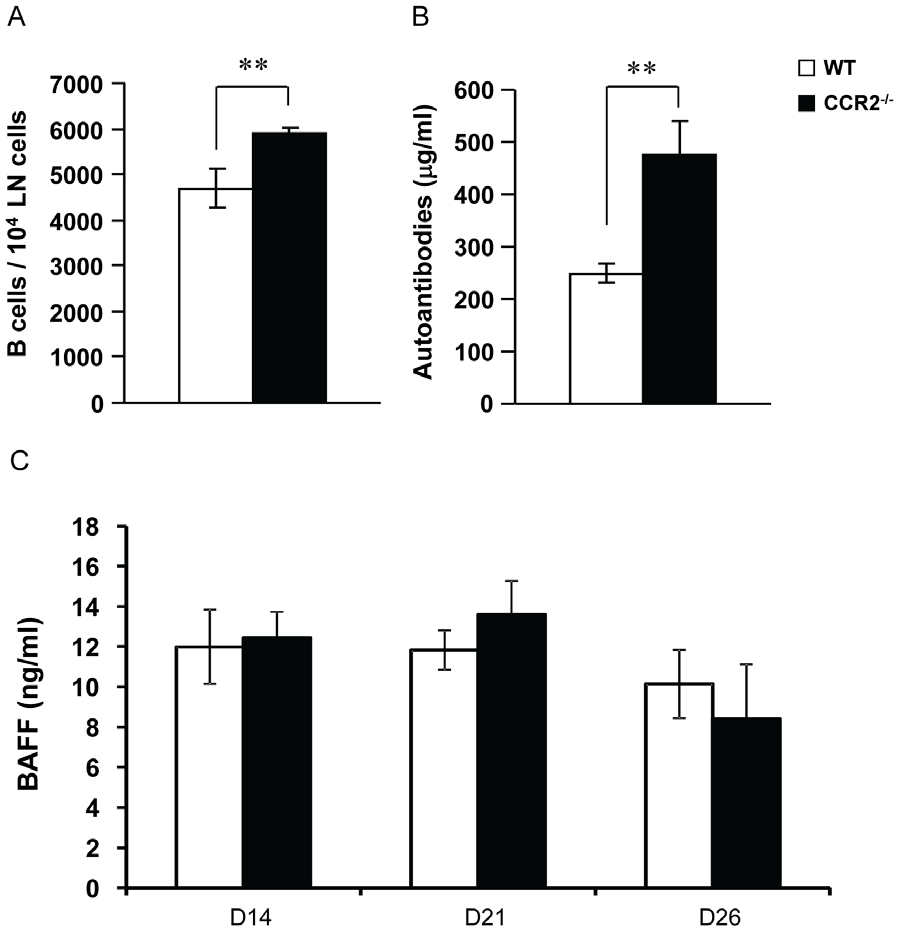
Antigen-specific antibodies increase with B cell expansion in CCR2^−/−^ mice. **A.** Single cell suspensions were isolated from the draining lymph nodes of mice at day 14 after immunization. B cells were identified as CD45R/B220^+^ cells by flow cytometry. Data shown are average numbers of B cells from WT (n = 6) and CCR2^−/−^ mice (n = 4), **p = 0.0004. **B.** Blood sera were collected from WT (n = 7) and CCR2^−/−^ mice (n = 8) at day 26 after immunization, and levels of anti-type II collagen antibodies (anti-CII) were determined by ELISA, **p = 0.005. **C.** ELISA results of serum levels of BAFF of WT and CCR2^−/−^ mice at day 14 (n = 4 vs. 5), day 21 (n = 7 vs. 8), and day 26 (n = 7 vs. n = 5) after immunization are shown.

IL-17 has been shown to act in synergy with B cell-activating factor (BAFF) to influence B cell biology in systemic lupus erythematosus [24]. Therefore we analyzed B cell abundance and their ability to produce type II collagen (CII)-specific antibodies in immunized CCR2^−/−^ mice. We found that CCR2^−/−^ mice have approximately 26% more B lymphocytes in the draining lymph nodes than that in WT mice (Figure 3A). In agreement with this result, significantly higher levels of anti-CII IgG were detected in the serum of these mice (Figure 3B). However, such increased B cell activities were BAFF-independent as we did not find significant BAFF elevation in CCR2^−/−^ mice (Figure 3C). Thus, the expansion of Th17 cells in CCR2^−/−^ mice appears to be associated with enhanced humoral immune responsiveness to type II collagen.

### Upregulated neutrophils in CCR2^−/−^ mice

**Figure 4:**
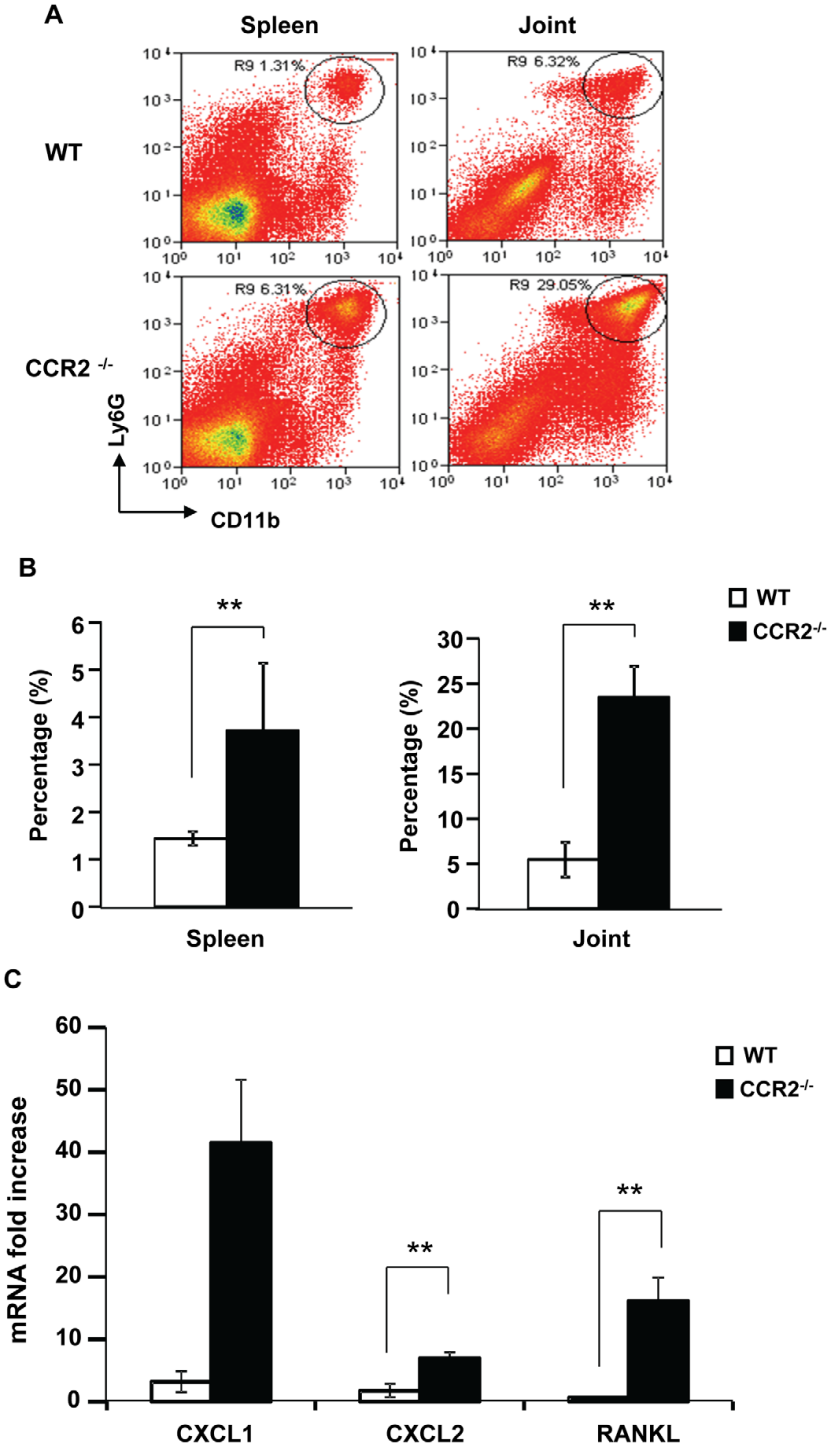
Neutrophil infiltration increases in the spleen and the joints of CCR2^−/−^ mice. Cells were isolated from the spleen and the hind paws of mice at day 48 after immunization and were stained for flow cytometry analysis. Ly6G^+^CD11b^+^ (Ly6C^+^) cells were gated as neutrophils. Shown are representative dot plots (**A**) and quantification of the number of neutrophils (**B**) of three experiments, **p = 0.02 in the spleen and 0.002 in the joints. **C.** Joint tissues were isolated from the hind paws of mice at day 48 after immunization. The expression of CXCL1, CXCL2, and RANKL mRNA was measured by real time PCR in WT (n = 5) versus CCR2^−/−^ (n = 4) mice, p = 0.059, 0.016, 0.026, respectively.

IL-17 promotes inflammation through stimulation of granulopoiesis *in vivo* [25]. To examine activities of neutrophils in immunized CCR2^−/−^ mice, we investigated the abundance of Ly6G^+^Ly6C^+^CD11b^+^ neutrophils in the spleen and the joints. In the spleen, we found substantially more neutrophils in CCR2^−/−^ mice than in WT mice (Figure 4A and B). Correspondingly, enhanced neutrophil infiltration into the joints was detected in these mice (Figure 4A and B). The expansion of neutrophils in CCR2^−/−^ mice was also observed in the histological joint sections with the H&E stain (Figure 1B). As neutrophil recruitment is driven by neutrophil-tropic chemokines, CXCL1 and CXCL2, we examined joint expressions of these cytokines. Indeed, these cytokines increased substantially in the joints of immunized CCR2^−/−^ mice (Figure 4C). In addition, increased expression of receptor activator of nuclear factor kappa-B ligand (RANKL) was also observed in the joint of CCR2^−/−^ mice (Figure 4C). These results indicate that Th17 cell expansion induced by CCR2 deficiency is associated with cellular responses in collagen-induced arthritis.

### Anti-IL-17A antibody treatment ameliorates CIA in CCR2^−/−^ mice

**Figure 5:**
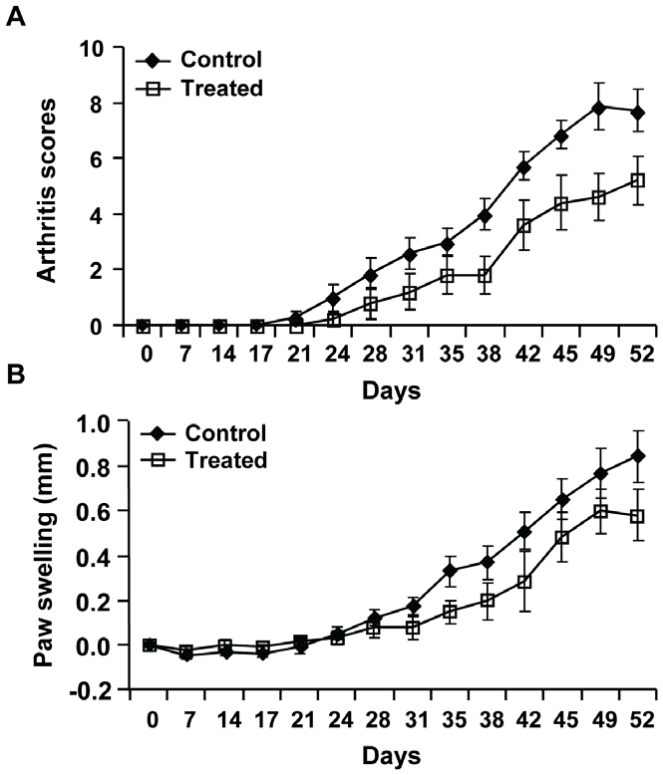
Treatment with anti-IL-17A antibodies reduces CIA in CCR2^−/−^ mice. CCR2^−/−^ mice were immunized with bovine type II collagen at day 0 and were treated 5 times in 2.5 weeks with either an anti-IL-17A antibody (Treated, n = 5) or an isotype control IgG1 (Control, n = 7) starting from day 14 after immunization. Arthritis score (A) and paw swelling (B) were recorded over time (Days). Data represent one of the two independent experiments performed. Differences of both arthritis scores and paw swelling during the course of disease between treated and control groups are significant (p<0.01).

To confirm a role of IL-17 in the pathogenesis of CCR2-mediated exacerbation of arthritis, we examined the effect of a murine anti-IL-17A antibody on the development of CIA in CCR2^−/−^ mice. As shown in Figure 5, CCR2^−/−^ mice that were treated with the anti-IL-17A antibody developed milder disease than CCR2^−/−^ mice that were treated with an isotype control antibody. In addition, the onset of CIA in the anti-IL-17A treated group was also delayed. These results indicate that IL-17A contributes, at least in part, to the exacerbation of collagen-induced arthritis in CCR2^−/−^ mice.

### Monocytes decrease in the spleen, but are abundant in the bone marrow and arthritic joints in immunized CCR2^−/−^ mice

**Figure 6:**
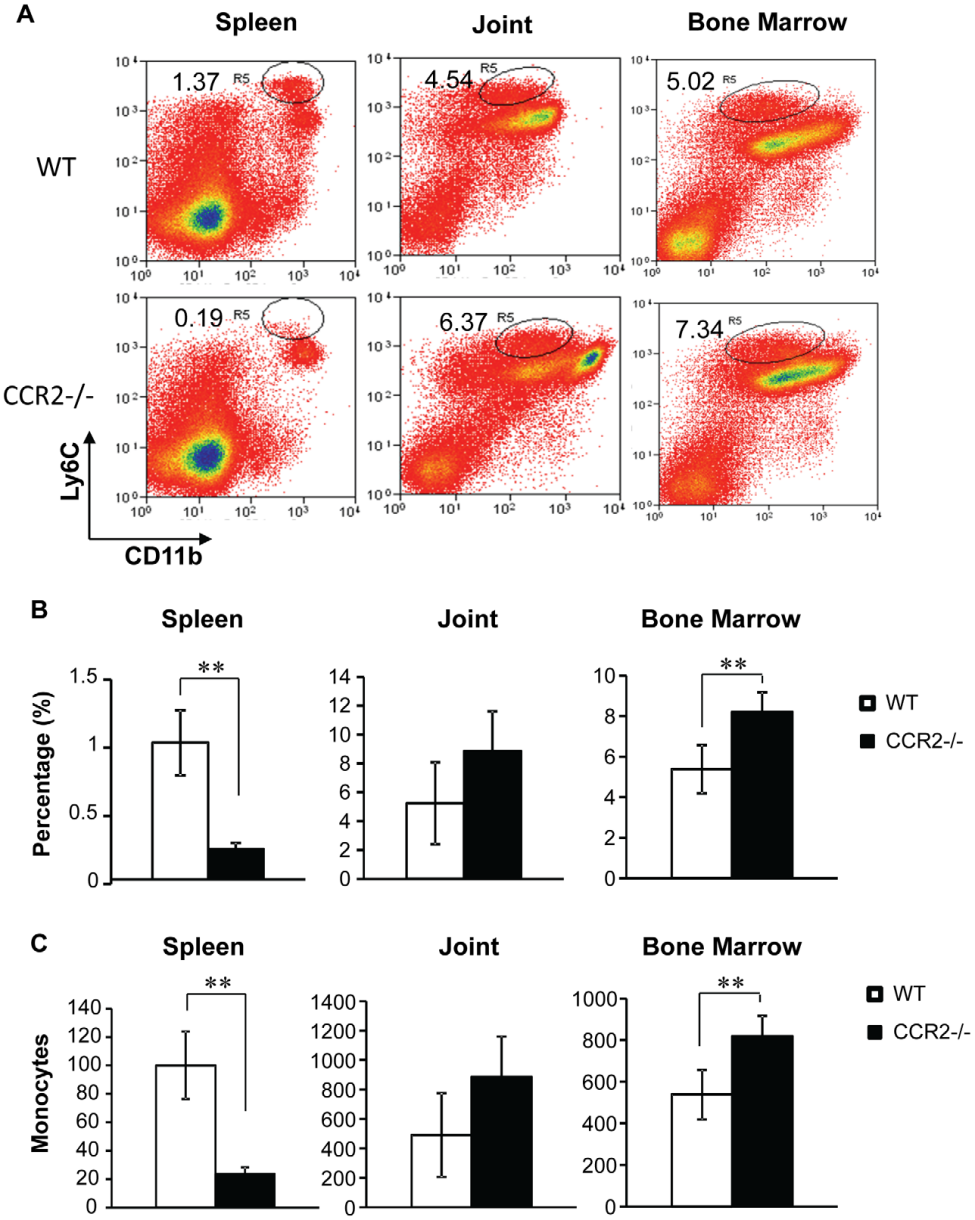
Monocytes decrease in the spleen, but are abundant in the bone marrow and arthritic joints in immunized CCR2^−/−^ mice. Single cell suspensions of the spleen, joints, and bone marrow from collagen-immunized mice were labeled with corresponding antibodies. The monocyte populations were recognized by flow cytometry as L6C^high^CD11b^+^Ly6G^−^ cells (A). The abundance of monocytes in each compartment was quantified as percentage (B) and number of monocytes per 10^4^ of total gated live cells (C) in immunized CCR2^−/−^ and WT mice. These data are results of at least three separated experiments, **p<0.05.

Given that CCR2 is highly expressed on monocytes [26] that have been recently identified as T cell suppressors under autoimmune conditions [27], we examined monocytes in immunized CCR2^−/−^ mice. We found that Ly6C^high^CD11b^+^ CCR2-expressing monocytes were almost absent from the spleen. In contrast, these monocytes were more abundant in the bone marrow and the inflamed joints in CCR2^−/−^ mice compared to WT mice. Together, these results indicate that CCR2 mediates monocyte immigration from the bone marrow while serving no role in monocyte recruitment to the arthritic joints (Figures 6A–C). These data also suggest that the lack of CCR2-expressing monocytes in CCR2^−/−^ mice in the spleen after immunization may promote Th17 cell polarization and proliferation in the priming phase of collagen induced arthritis.

## Discussion

CCR2 is a chemokine receptor predominantly expressed on monocytes and is considered proinflammatory in response to inflammation. However, CCR2 deficiency unexpectedly induces severe autoimmune arthritis with accelerated disease onset; and the underlying mechanism is not completely understood. In this study, we show that Th17 cells are selectively expanded prior to disease onset in the draining lymph nodes in collagen-immunized CCR2^−/−^ mice compared to WT controls. Consistently, augmented IL-17A, IL-6, and IL-1β levels are observed in the blood and in the inflamed joints of CCR2^−/−^ mice, which are accompanied by enhanced autoantibody production and neutrophil infiltration. Neutralizing IL-17A has an ameliorating effect on the severe arthritis observed in CCR2^−/−^ mice. Our results suggest a previously unrecognized mechanism that CCR2 may be important in maintaining immunological homeostasis and protecting against collagen-induced arthritis via regulation of Th17 cell responses. Given that monocytes suppress T cell function during autoimmunity, our finding that monocytes are substantially decreased in the spleen but not in the bone marrow and the arthritic joints in CCR2^−/−^ mice may provide a biological link between CCR2 and Th17 cells during the pathogenesis of collagen-induced arthritis.

Accumulated evidence has implicated an important role for Th17 cells in autoimmune disease such as RA [28], [29], [30]. Th17 cell differentiation from naïve CD4+ T cells depends on IL-6 and TGF-β production. Given that TGF-β is also required for Treg cell generation and IL-6 can reverse Treg cell-mediated suppression of autoreactive T cells [31], IL-6 is an essential differentiation factor for Th17 polarization. On the other hand, IL-17 activates cells in synovium, such as synovial fibroblasts and monocytes/macrophages, to produce IL-6, IL-1 and TNF-α and thus sustain joint inflammation [32], [33], [34]. Consistent with these findings, our data show that early Th17 cell expansion in CCR2^−/−^ mice is accompanied by elevated IL-6 levels in the blood. Increased levels of IL-17A expression are also observed together with augmented IL-6 and IL-1β abundance in the arthritic joints of CCR2^−/−^ mice. Additionally, RANKL expression is significantly up-regulated in CCR2-deficient joints, which is consistent with findings that joint IL-17A stimulates osteoclasts to express RANKL that promotes bone erosion [35], [36]. Together, our results support the hypothesis that a positive feedback loop between Th17 cells and IL-6 is a key factor involved in tolerance breakdown and tissue injury in autoimmune arthritis. Our data further suggest a potential beneficial role of CCR2 in this process.

Th17 cells promote autoimmune pathology through effects on cellular and humoral immune responses. Indeed, increased Th17 cells are not only accompanied by a substantial expansion of neutrophils in both the spleen and joints but also B cell up-regulation with a consequent elevation of type II collagen-specific antibodies in immunized CCR2^−/−^ mice. IL-17A is known to stimulate granulopoiesis *in vivo* [25] and stimulates neutrophil-specific chemokines such as MIP-2 and IL-8 [37]. Blockade of IL-17A in IFN-γ receptor knockout mice with CIA ameliorates joint inflammation and bone erosion by significant reductions of the splenic expansion and joint influx of neutrophils [38], suggesting a critical role of IL-17-stimulated neutrophils in autoimmune pathology. IL-17 receptor is expressed on B cells, and IL-17 has been shown to work together with B cell-activating factor to control the survival and differentiation of B cells into antibody producing cells in autoimmunity [24], [39]. In addition, IL-17 is found to drive autoimmune responses by promoting the formation of spontaneous germinal centers [40], and IL-17A-deficient mice have decreased autoantibodies in CIA and EAE [7], [41]. These results provide rationales for our data that increased Th17-cell responses contribute to the exacerbated arthritis in CCR2^−/−^ mice.

Recently, Fuji et al. have published a well-designed study showing that ablation of the Ccr2 gene exacerbated polyarthritis in IL-1 receptor antagonist-deficient (Il1rn^−/−^) mice [42]. Increased neutrophil accumulation and IL-I7 production were observed in the inflamed joints of those double knockout mice. Neutralizing CXCR2, a neutrophil chemokine receptor, reduced arthritis severity in Il1rn^−/−^Ccr2^−/−^ mice. Since our collagen-induced arthritis model is different from the Il1rn-dependent model in which polyarthritis develops spontaneously [43], it is difficult to compare directly which therapeutic method, anti-IL-17A or anti-CXCR2 is more effective. It is possible that the augmented neutrophil infiltration into the joints in CCR2^−/−^ mice contribute to the aggravated disease process through an additional Th17-independent mechanism. However, since neutrophils are key innate cells against infection, targeting such cells for the treatment of chronic disease like RA needs to be done cautiously.

Monocytes traffic to tissues and differentiate into macrophages or dendritic cells in response to inflammation [44], [45]. Recent evidence shows that CCR2 does not mediate monocyte trafficking into the inflamed tissues [46], [47], but is rather involved in the egress of monocytes from the bone marrow [46], [48]. Indeed, monocytes are almost absent from the spleen, but are abundant in the bone marrow and inflamed joints in immunized CCR2^−/−^ mice. Intriguingly, CCR2-expressing monocytes have been recently shown to have an immune regulatory role via suppression of T cell activities under tumor and autoimmune conditions [27], [49]. It is conceivable that CCR2 deficiency alters T-cell suppressive monocytes, which results in Th17 cell expansion. CCR2 is also expressed on a subset of Treg cells that inhibit T cell proliferation *in vitro* [50]. However, we find similar numbers of Treg cells between immunized CCR2^−/−^ and WT mice, suggesting that upregulated Th17 cells in CCR2^−/−^ mice are Treg-cell-independent. But our data cannot exclude the possibility that a small subset of Treg cells is affected by CCR2 deficiency.

Although CCR2 is a drug target for the treatment of some inflammatory conditions, blocking CCR2 or deleting Ccr2 gene worsens autoimmune arthritis. Our data show that Th17 cell dysregulation is responsible, at least in part, for the exacerbated phenotype in CCR2-deficient mice. CCR2 may have a protective role against autoimmune arthritis. Neutralization of IL-17 is a promising therapeutic approach against autoimmune arthritis possibly by inhibiting the excessive joint recruitment of neutrophils and monocytes as local injection and expression of IL-17 promote migration of both cell types to the joints [51], [52].
